# Successful Non-surgical Management of Emphysematous Hepatitis During Chemotherapy for Acute Myeloid Leukemia

**DOI:** 10.7759/cureus.91222

**Published:** 2025-08-29

**Authors:** Maiya Chen, Kazuhide Takata, Yuta Nakashima, Yasushi Isobe, Yasushi Takamatsu

**Affiliations:** 1 Department of Respiratory Medicine, Fukuoka University Hospital, Fukuoka, JPN; 2 Department of Gastroenterology and Medicine, Fukuoka University Faculty of Medicine, Fukuoka, JPN; 3 Division of Medical Oncology, Hematology and Infectious Disease, Department of Internal Medicine, Fukuoka University Faculty of Medicine, Fukuoka University Faculty of Medicine, Fukuoka, JPN

**Keywords:** acute myeloid leukemia, antibiotic therapy, emphysematous hepatitis, liver infectious disease, non-surgical management

## Abstract

Emphysematous hepatitis (EH) is a rare and fatal liver infection characterized by gas replacement of the liver parenchyma. We report a case of a 69-year-old male who was admitted for chemotherapy for acute myeloid leukemia (AML). On admission, he presented with fever and right hypochondrial pain, and EH was diagnosed by computed tomography (CT). Although invasive procedures were withheld due to thrombocytopenia, the patient was successfully treated with antibiotics alone. EH may be managed conservatively if diagnosed at an early stage.

## Introduction

Emphysematous hepatitis (EH) is a rare and fatal infection characterized by necrotic gas formation within the hepatic parenchyma. Since it was first reported by Blachar et al. in 2001 [1], the number of cases has remained limited, occurring primarily in patients with diabetes mellitus or a history of abdominal surgery. The diagnosis heavily relies on computed tomography (CT) and is distinguished from gas-forming liver abscesses by the absence of liquefactive abscess formation [2]. Due to the rapid progression of hepatic necrosis caused by gas-producing bacteria, the prognosis is extremely poor despite aggressive treatment. There is no consensus on optimal treatment, but aggressive surgical intervention, in addition to antibiotic therapy and percutaneous drainage, is considered important [3,4]. We report a case of EH successfully managed with prompt antibiotic therapy without surgical interventions.

## Case presentation

A 69-year-old male was hospitalized for chemotherapy to treat acute myeloid leukemia (AML). He had a history of follicular lymphoma and diffuse large B-cell lymphoma, treated successfully 10 years prior with chemotherapy and autologous transplantation. One year before admission, he was diagnosed with AML and began treatment with venetoclax plus azacitidine (VEN+AZA) therapy. Neutropenia (CTCAE v5.0 Grade 3-4) persisted until three months before admission. He was admitted for the third course of VEN+AZA therapy.

The patient had no history of diabetes mellitus or abdominal surgery. Upon admission, the patient was alert, with vital signs showing a temperature of 38.8 ℃, heart rate of 110 beats per minute, and blood pressure of 122/72 mmHg. Physical examination revealed mild right hypochondrial pain without jaundice or peritoneal irritation. Laboratory findings showed a white blood cell count of 7,800/μL, neutrophils 1,372/μL (Grade 2 neutropenia), platelets 34,000/μL (Grade 3 thrombocytopenia), C-reactive protein 21.6 mg/dL, procalcitonin 11.2 ng/mL, and elevated liver enzymes (total bilirubin 1.9 mg/dL, aspartate aminotransferase 102 U/L, alanine aminotransferase 101 U/L, alkaline phosphatase 170 U/L, gamma-glutamyl transpeptidase 82 U/L) (Table 1).

**Table 1:** Laboratory data on admission WBC, white blood cell count; RBC, red blood cell count; Hb, hemoglobin; Plt, platelets; TP, toral protein; ALB, albumin; T-Bil, toral-bilirubin; AST, aspartate aminotransferase; ALT, alanine aminotransferase; γ-GTP, gamma-glutamyl transpeptidase; ALP, alkaline phosphatase; CRP, C-reactive protein; Glu, glucose; BUN, blood urea nitrogen; Cr, creatinine; PCT, procalcitonin; PT, prothrombin time; APTT, activated partial thromboplastin time; Fbg, fibrinogen; FDP, fibrin/fibrinogen degradation products; IgG, Immunoglobulin G; IgA, Immunoglobulin A; IgM, Immunoglobulin M

Test	Value	Reference range
WBC (/μL)	7,800	3,300-8,600
RBC (10^4/μL)	253	435-555
Hb (g/dL)	7.6	13.7-16.8
Plt (10^3/μL)	34	158-348
Neut (%)	17.6	40-60
Lympho (%)	24.4	20-40
Mono (%)	57.4	2.0-8.0
Eosino (%)	0.5	1.0-4.0
Baso (%)	0.1	0.0-1.0
TP (g/dL)	7.5	6.0-8.3
ALB (g/dL)	3.4	3.8-5.3
T-bil (mg/dL)	1.9	0.2-1.2
AST (U/L)	102	13-30
ALT (U/L)	101	10-42
γ-GTP (U/L)	82	10-70
ALP (U/L)	170	44-147
CRP (mg/dL)	21.6	<0.15
Glu (mg/dL)	126	70-100
BUN (mg/dL)	14	8.0-20
Cr (mg/dL)	0.77	0.65-1.07
PCT (ng/mL)	11.2	<0.1
PT (INR)	0.98	0.8-1.1
APTT (sec)	36.8	25-35
Fbg (mg/dL)	479	200-400
FDP (μg/mL)	8	<10
IgG (mg/dL)	1,728	870-1,700
IgA (mg/dL)	323	110-410
IgM (mg/dL)	83	33-190

**Figure 1:**
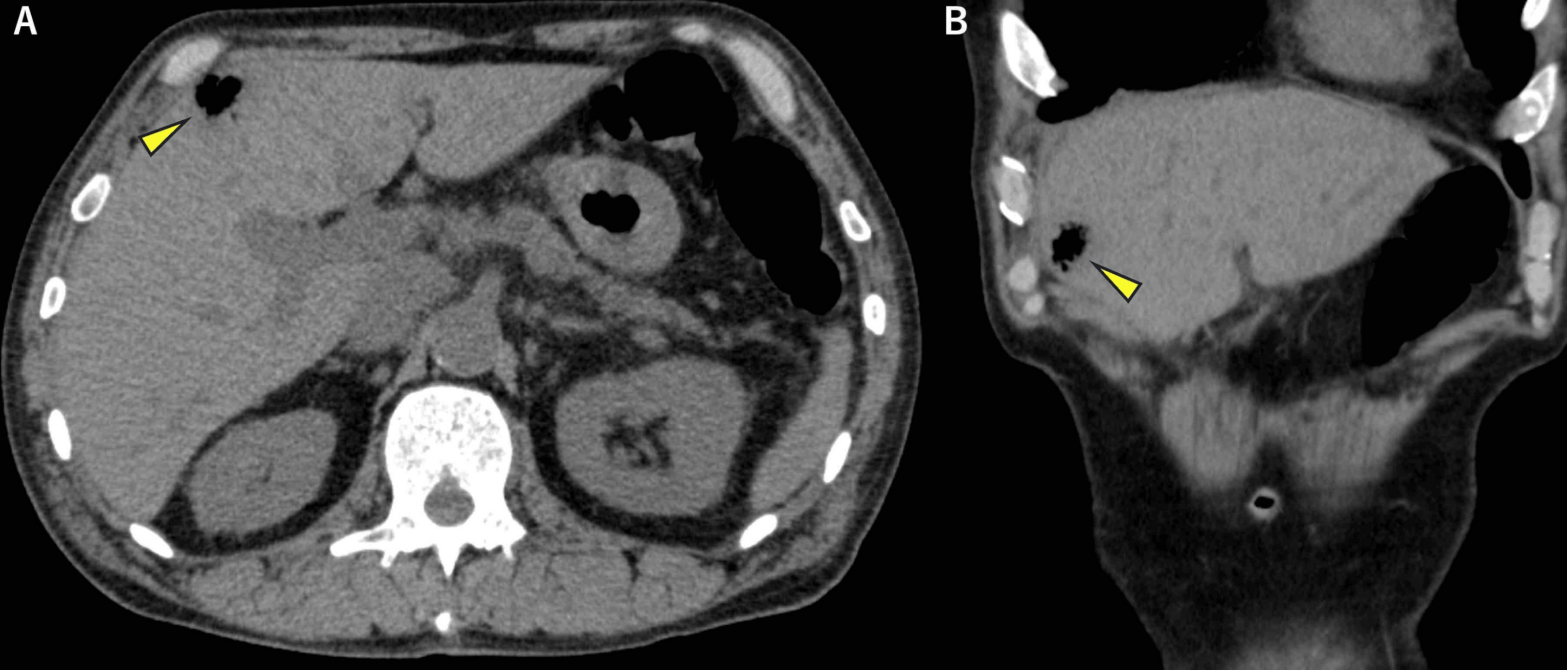
Abdominal non-contrast CT (A, B) Abdominal non-contrast CT revealed gas formation in liver segment Ⅳ, measuring up to 17 mm in diameter (arrowheads).

**Figure 2:**
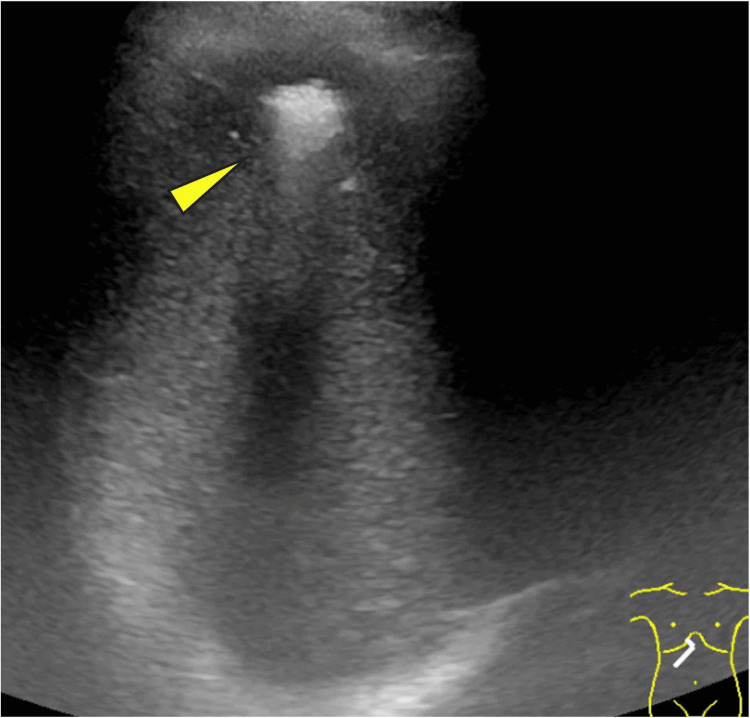
Abdominal ultrasound Abdominal ultrasound demonstrated a hyperechoic area suggestive of emphysematous change with no evidence of abscess formation (arrowhead).

Abdominal plain CT revealed gas formation in liver segment IV, measuring up to 17 mm in diameter (Figure 1). Abdominal ultrasonography (US) also showed an irregular hyperechoic area at the same location (Figure 2). However, no hypoechoic area suggestive of abscess collection was observed around the lesion, leading to a diagnosis of EH. All the examinations were performed on the day of admission, and the diagnosis of EH was confirmed on the same day.

**Figure 3:**
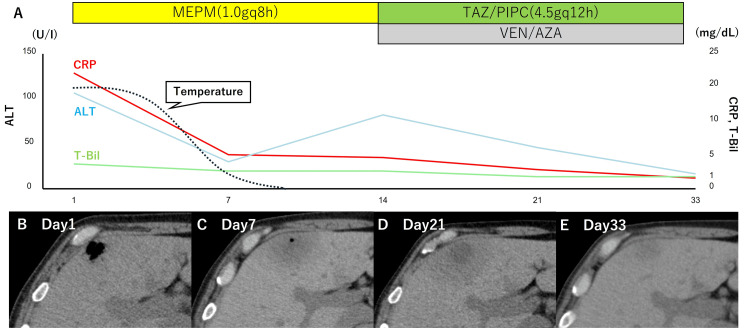
Clinical course (A) After the initiation of antibiotic therapy, the patient’s fever subsided, and inflammatory markers and liver enzymes gradually improved. (B-E) Serial CT imaging also showed progressive resolution of the emphysematous changes. MEPM, Meropenem; TAZ/PIPC, Tazobactam/Piperacillin; VEN/AZA, Venetoclax/Azacitidine; white blood cell count; T-Bil, toral-bilirubin; ALT, alanine aminotransferase; CRP, C-reactive protein

The clinical course is shown in Figures 3A-3E.

Following diagnosis, antibiotic therapy with meropenem (1.0gq8h) was promptly initiated. Due to Grade 3 thrombocytopenia, and considering the patient's stable condition and relatively small size of the hepatic lesion, percutaneous drainage and surgical interventions were initially withheld. The patient's fever subsided four days after starting antibiotic therapy, with hepatobiliary enzymes and CRP also showing a decreasing trend. On day 14 after diagnosis, the antibiotic was changed to tazobactam-piperacillin (4.5gq12h), considering the need to maintain anaerobic coverage and the prolonged Grade 4 neutropenia. On the same day, considering the favorable treatment course of EH, VEN+AZA therapy for AML was resumed. CT findings showed that the emphysematous lesions had almost completely disappeared after three weeks from onset. The patient died due to AML progression four months after the EH diagnosis; no EH recurrence was observed during the course of AML treatment.

## Discussion

EH is a rare and highly lethal gas-forming liver infection. Porez et al. reported that 10 out of 14 patients (71%) developed hemodynamic failure within 24 hours of hospital admission [5]. To our knowledge, 25 cases have been reported to date [1-14]; 10 of these patients survived [2-11]. While all these cases required percutaneous drainage or surgical resection, our case was successfully treated with antibiotic therapy alone (Table 2).

**Table 2:** Previously reported cases of surviving EH including our case EH, emphysematous hepatitis

Reference	Age/Sex	Medical history	Imaging	Treatment	Pathogens
Ramalho et al. (2017) [8]	78/F	-	Gas collection in liver segments Ⅱ and III	Left hepatic lobectomy, Antibiotics	Enterococcus faecium
Ghosn et al. (2019) [3]	38/F	Diabetes mellitus, cholecystectomy, and ventral hernia repair	Mixed collection containing air and debris mainly in liver segments Ⅱ and IV (80×70×55mm)	Rapid surgical debridement, Antibiotics	*Escherichia coli* *Enterococcus faecium*
Ferrero et al. (2021) [7]	67/F	-	Abundant air in liver segments VI, VII, and VIII	Urgent surgery, including drainage, Debridement, Antibiotics	Escherichia coli
François et al. (2022) [2]	70/F	Diabetes mellitus, cholecystectomy, and heterozygote alpha-1 antitrypsin deficiency	Air-filled cavity in the right liver lobe (90mm)	Percutaneous drainage, ERCP with sphincterotomy of the papilla of Vater, Antibiotics	Escherichia coli Streptococcus anginosus Klebsiella oxytoca
Pan et al. (2023) [6]	48/M	Diabetes mellitus and hypertension	Air collection with some remaining parenchyma debris mainly in liver segments II and IV (66 × 55 × 53mm)	Percutaneous drainage, Antibiotics	Klebsiella oxytoca
						Porez et al. (2023) [5]	59/M	Diabetes mellitus and hypertension	Multiple gas bubbles without an enhancement wall or collection in liver segment VIII (58 × 50mm)	Ultrasound-guided drainage, Median laparotomy, Placement of a Pezzer drain	Klebsiella pneumoniae
Bayerl et al. (2023) [4]	79/M	Hypertension, hyperlipidemia, diabetes mellitus, benign prostate hyperplasia, atrial fibrillation, and chronic heart failure	Gas collection in liver segment VI (34mm)	Surgical debridement, Atypical liver resection, Insertion of a T-drain, Antibiotics	Clostridium perfringens Enterococcus faecium
El-Faouri et al. (2025) [9]	55/F	Diabetes mellitus	A large heterogeneous gas density in the liver (90 × 60mm)	Percutaneous drainage, Surgical debridement, partial liver resection of segment Vl, Antibiotics	-
Chen et al. (2025) [10]	57/M	Diabetes mellitus	Gas in the right liver lobe (86 × 65mm)	Percutaneous drainage, Antibiotics	Klebsiella pneumoniae
Paida et al. (2025) [11]	81/F	Ischemic cerebrovascular disease, hypertension, and dyslipidemia	Gas in the right liver lobe (involving multiple liver segments)	Percutaneous drainage, Antibiotics	Escherichia coli
Our case	69/M	Acute myeloid leukemia	Gas collection in liver segment IV (17mm)	Antibiotics (meropenem 14 days, tazobactam-piperacillin 14 days)	-

Risk factors for EH include poorly controlled diabetes mellitus, prior abdominal surgery, and gastrointestinal cancers [3,5]. Our patient had none of these but was immunocompromised from leukemia treatment with prolonged Grades 3-4 neutropenia, which may have predisposed to EH development. Common causative organisms of EH include *Escherichia coli*, *Klebsiella pneumoniae*, *Klebsiella oxytoca*, *Enterococcus faecium*, *Enterobacter species*, *Pseudomonas species*, *Clostridium perfringens*, and *Streptococcus species* [1,3,5]. These gas-producing bacteria ferment under hyperglycemic or ischemic conditions, rapidly accumulating hydrogen and carbon dioxide in hepatic tissue and accelerating necrosis [2,3,5].

No established diagnostic criteria exist for EH; diagnosis primarily relies on imaging studies, particularly CT, which offers high sensitivity and specificity. EH is distinguished from gas-forming liver abscesses by confirming emphysematous changes without liquefactive abscesses [2,4,5]. In our case, the initial lesion size was 17mm, smaller than those reported in other cases (Table 2), suggesting that our diagnosis was made at an early stage.

Upon confirmation of the diagnosis, empirical antibiotic therapy with meropenem--a broad-spectrum carbapenem--was promptly initiated, given the high lethality associated with EH. Although blood cultures yielded negative results and antimicrobial susceptibility could not be determined, the patient exhibited defervescence and a marked reduction in inflammatory markers. Based on the clinical response and the need to maintain anerobic coverage, the antibiotic regimen was subsequently transitioned to tazobactam-piperacillin, a combination of a β-lactam antibiotic and a β-lactamase inhibitor. While no established treatment protocol exists for EH, previous reports have emphasized the importance of early percutaneous drainage and surgical intervention, in addition to antibiotic therapy and strict glycemic control [3-5]. All reported surviving cases, except our case, underwent percutaneous drainage and/or surgical resection (Table 2).

In our case, percutaneous drainage and surgical interventions were initially withheld due to persistent Grade 3 thrombocytopenia. However, since the condition was diagnosed on admission, prompt antibiotic therapy led to a favorable outcome. Even in patients who are immunocompromised or for whom surgical interventions are high-risk, like our case, early diagnosis may enable successful conservative management of EH.

Our case has several limitations. First, negative blood cultures precluded definitive identification of the causative pathogen. Second, although this case showed no obvious abscess on ultrasound, the CT scan was performed without contrast enhancement, which limited the evaluation of the wall structure and liquefaction. Additionally, the possibility that severe, prolonged neutropenia precluded abscess formation should also be considered. Third, in this case, percutaneous drainage was not actively performed due to thrombocytopenia; however, temporary elevation of platelet counts through platelet transfusion and/or thrombopoietin receptor agonists might have enabled safe percutaneous drainage. If percutaneous drainage had been performed, earlier remission might have been achieved.

Future discussions on the management of EH should consider diverse strategies tailored to disease severity. Identifying patients who respond well to conservative treatment is important for developing individualized treatment plans. Like our case, cases that are diagnosed early and have relatively small lesions may be cured with antibiotics alone. Furthermore, in cases where the patient is hemodynamically stable and has not progressed to sepsis, a strategy of prioritizing antibiotics and percutaneous drainage, with prompt surgical intervention when necessary, is likely to be an effective strategy. While further case accumulation is needed, our report will serve as a new contribution to the consideration of treatment strategies for EH.

## Conclusions

This case demonstrates that, in select EH patients diagnosed early with small lesion size and hemodynamic stability, conservative treatment with antibiotics alone may be effective, even in immunocompromised hosts. While EH remains a life-threatening infection, individualized treatment strategies based on lesion size, patient stability, and comorbid conditions are warranted.
